# Electrically evoked compound action potential artifact rejection by independent component analysis: Technique validation^☆^

**DOI:** 10.1016/j.heares.2013.04.005

**Published:** 2013-08

**Authors:** Idrick Akhoun, Colette M. McKay, Wael El-Deredy

**Affiliations:** School of Psychological Sciences, Faculty of Medical and Human Sciences, The University of Manchester, Manchester M13 9PL, UK

**Keywords:** AN, auditory nerve, CI, cochlear implant, CI24RE, Cochlear^®^ Nucleus Freedom™ cochlear implant – 22 active electrodes and 2 ground electrodes, ECAP, electrically-evoked compound action potential, ECAP-FM, ECAP obtained with the forward-masking technique, ECAP-ICA, ECAP obtained with the ICA artifact rejection technique, ICA, independent component analysis, IC, independent component (or source), JADE-R, joint approximate diagonalisation of the cross-cumulants eigenmatrices (computational implementation of ICA), MP1, extracochlear ground electrode, MP2, ground electrode placed on the case of the cochlear implant, N1P1, peak-to-peak voltage difference measurement of ECAP amplitude, RMS, root mean square, SNR, signal to noise ratio

## Abstract

The electrically-evoked compound action potential (ECAP) is the synchronous whole auditory nerve activity in response to an electrical stimulus, and can be recorded in situ on cochlear implant (CI) electrodes. A novel procedure (ECAP-ICA) to isolate the ECAP from the stimulation artifact, based on independent component analysis (ICA), is described here. ECAPs with artifact (raw-ECAPs) were sequentially recorded for the same stimulus on 9 different intracochlear recording electrodes. The raw-ECAPs were fed to ICA, which separated them into independent sources. Restricting the ICA projection to 4 independent components did not induce under-fitting and was found to explain most of the raw-data variance. The sources were identified and only the source corresponding to the neural response was retained for artifact-free ECAP reconstruction. The validity of the ECAP-ICA procedure was supported as follows: N_1_ and P_1_ peaks occurred at usual latencies; and ECAP-ICA and artifact amplitude-growth functions (AGFs) had different slopes. Concatenation of raw-ECAPs from multiple stimulus currents, including some below the ECAP-ICA threshold, improved the source separation process. The main advantage of ECAP-ICA is that use of maskers or alternating polarity stimulation are not needed.

## Highlights

• Novel technique to cancel artifact in electrically-evoked compound action potentials.
• No maskers or alternate polarity are required.
• ECAPs output from the procedure are compared to forward-masking technique.

## Graphical abstract

**Figure:**
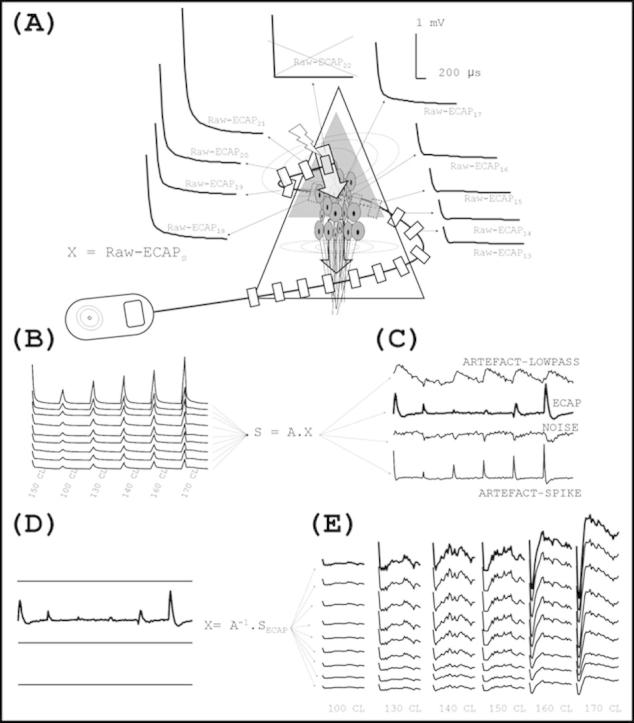

## 1 Introduction

This paper presents a novel artifact rejection procedure for electrically-evoked compound action potentials (ECAPs) based on Independent Component Analysis (ICA). This new method, denoted ECAP-ICA, avoids the use of masker pulses or alternating polarity stimulus pulses.

### 1.1 Artifact rejection in ECAP measurements

ECAPs reflect the synchronous whole auditory nerve (AN) response to electrical stimulation. ECAPs are routinely used in clinics to objectively measure the functionality of auditory nerve activation. A stimulation artifact results from a voltage decay following the biphasic current pulse. The artifact waveform is usually several orders of magnitude larger than the ECAP and is a decaying exponential with a time constant of several hundreds of microseconds, which is sufficiently long to overlap with the neural response. Two main artifact cancellation methods are available in clinical software: the use of alternating stimulus polarity, or forward-masking (ECAP-FM).

**Fig. 1:**
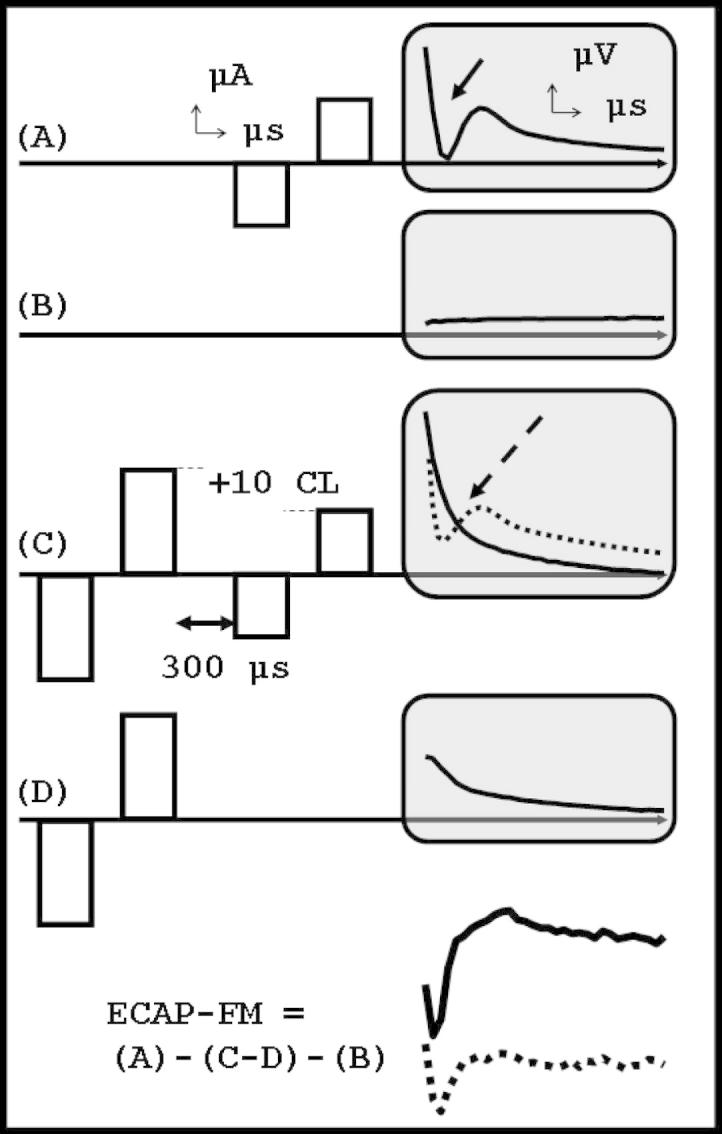
Forward-masking ECAP artifact subtraction paradigm (ECAP-FM). Each buffer (grey windows) is an average of fifty consecutive recordings. Buffer A (referred to as raw-ECAP) is the superposition of ECAP elicited by the probe biphasic electrical pulse, indicated by an arrow, and the probe stimulation artifact. Buffer C records the probe artifact and no probe ECAP, along with the remaining masker artifact and masker ECAP. If the masker fails to render the auditory nerve completely refractory, a residual probe response remains (dashed line). Buffer D records the influence of the masker alone, and buffer B records the amplifier switch-on effect. The ECAP-FM revealed by subtraction is plotted in the bottom: if incomplete masking occurs in buffer C, the ECAP-FM is distorted and underestimated due to subtraction of the residual ECAP response.

As shown schematically in Fig. 1, four buffers are recorded in the forward masking method. On buffer C, a preceding masker-pulse is used to set the auditory nerve in a refractory state and therefore only the probe artifact is recorded, along with any remaining masker artifact and response. The artifact is obtained by subtracting the effects of the masker alone (buffer D) from buffer C. ECAP is finally obtained by subtracting the artifact (buffers C-D) from buffer A (ECAP and artifact) from which the effect of amplifier-switch-on (buffer B) has been subtracted. Hence, the subtraction (A)-(C-D)-(B) results in ECAP-FM.

The alternating polarity method requires two buffers to be recorded and summed together: one resulting from a cathodic-first biphasic pulse and the other resulting from an anodic-first biphasic pulse. It is assumed that the artifacts in the two cases are exactly equal amplitude but opposite polarity and hence cancel each other, and that the neural responses to both polarity pulses are identical, making their sum equal to the ECAP with double the amplitude.

Both of these methods rely upon physiological assumptions that are known to be only approximately true. In the case of forward masking, all of the auditory nerve fibers may not be in a refractory state when the probe stimulus follows the masker, leading to a partial probe ECAP that is subsequently subtracted from the probe ECAP in buffer A (Brown et al., 1990 -see also dashed waveform on buffer C in Fig. 1). In the alternating polarity method, anodic-first and cathodic-first biphasic stimulus pulses do not generate the same auditory nerve activity: the ECAPs have different latencies and amplitudes, resulting in distorted ECAPs after addition (Miller et al., 2000). Moreover, our own measurements in saline suggested that stimulation artifacts are not exactly equal and opposite for the two polarities, leading to a substantial residual artifact in the final ECAP. Two recent signal processing studies have been conducted to enhance traditional alternating polarity and forward-masking techniques (Alvarez et al., 2007, 2008). For both alternating polarity and forward-masking, the recorded buffers were weighted before subtraction in order to result in ECAP waveforms as close as possible to the description of a good ECAP waveform (as defined by clinical visual observation). In these studies, a very large ECAP database was used, in which each ECAP waveform was rated by expert audiologists. However, it is unclear how to relate these weighting coefficients to physiological or physical phenomenon.

A third artifact rejection technique called ‘precision-triphasic pulse artifact rejection technique’ (Bahmer et al., 2010), uses a triphasic pulse with a small portion of the charge of the first phase (around 10%) allocated to a third phase. This method, like ECAP-ICA, avoids the use of masker pulses or alternating polarity. However, triphasic pulses induce a different excitation pattern than clinically-used biphasic pulses. A fourth technique was introduced by Klop et al. (2004) who used an electrical amplifier with a compensation circuit at the input to reduce the residual stimulation artifact by electrical subtraction, and found they could reduce the time course of the artifact from around 200 μs to less than 30 μs. This technique has not yet been implemented in clinical cochlear implant (CI) settings.

### 1.2 Independent component analysis: a denoising technique for ECAP artifact cancellation

ICA is a blind-source-separation technique based on higher-order statistics that aims to separate independent sources from linear mixtures recorded on different sensors (for computational details see e.g. Comon, 1994; Bell and Sejnowski, 1995; Cardoso, 1999; Hyvarinen and Oja, 2000). It should be noted that no *a priori* knowledge is required about the sources. ICA decomposes the recordings into sources that are maximally statistically independent. The ICA rationale applies the central limit theorem that stipulates that the more independent the sources in a mixture, the more Gaussian the mixture's probability density function: the less Gaussian a variable's distribution is, the more independent it is assumed. Gaussianity is measured by kurtosis: zero kurtosis implies a Gaussian distribution. After ICA, it is then up to the experimenter to interpret these sources as relevant physical phenomenon. Finally, the position and number of sensors available are important parameters for ICA success: ICA demands at least the same number of sensors as the expected sources (Hyvarinen et al., 2001). More sensors than sources may help ICA to determine the independent relationship between the sources; however additional sensors may be redundant.

ICA has been used to separate artifact in cochlear implant cortical recordings (Gilley et al., 2006; Castaneda-Villa and James, 2011; Viola et al., 2011). In these studies, ICA was applied to multi-channel recordings of cortical potentials from scalp electrodes. The CI stimulation induces a large artifact described as an artifact pedestal followed by an overshoot period that can overlap with the target cortical response. The ICA approach requires simultaneous recording of artifact + cortical response mixtures at different locations on the scalp. However, current ECAP recording technology allows recordings to be made on only one intracochlear electrode at a time. Thus, in the ECAP-ICA method described here, ICA was applied to multiple intracochlear telemetry recordings of the ECAP + artifact + noise mixture (raw-ECAPs) obtained sequentially on different recording electrodes. It was assumed that the physical phenomena generating the electrical stimulation artifact and the ECAP would be exactly the same for each sequential recording, as they would be in simultaneous recordings. Artifact, ECAP, and noise separation is theoretically possible provided that those signals behave independently from each other. To verify that ECAP-ICA was a genuine physiological auditory nerve response and was successfully separated from the artifact, the following criteria were applied:

1. ECAP-ICA waveforms should have the typical N1-P1 pattern, with peak latencies in the range of those obtained by other methods such as ECAP-FM. The ECAP-ICA amplitudes should be broadly consistent with the range of ECAP amplitudes reported in the literature.
2. ECAP amplitude should increase with stimulus level at a different rate to the artifact amplitude. If sufficiently low stimulus levels are used, ECAP amplitude should reach zero (ECAP threshold) at a stimulus level for which the stimulus artifact is still evident.
3. ECAP and artifact amplitudes should change across recording-electrode sites in different ways.

## 2 Material and methods

### 2.1 Subjects

Eight adult CI24RE Nucleus Freedom™ (Cochlear Ltd.^®^) cochlear implant recipients participated in this study. The study was conducted under the approval of the NHS Ethics Committee. Details of these subjects are contained in Table 1.

### 2.2 Equipment, stimulation and recording parameters

All raw-ECAPs (that is, the mixture of artifact, response and noise), were recorded using Custom Sound EP hardware and software, with stimuli controlled via a Freedom processor, and responses obtained via Neural Response Telemetry (NRT). Recording parameters are listed below. Data from the buffers were downloaded into spreadsheets for MATLAB analysis.

Stimuli were charge-balanced biphasic pulses in monopolar mode (using a given intracochlear electrode (active) and the extra-cochlear MP1 electrode (reference)). Electrodes 14 (middle of the array), 17, and 22 (apical tip of the array) were activated with cathodic-first pulses, and electrode 17 was also activated with anodic-first pulses. For some subjects a subset of these conditions was used, or electrode 21 instead of 22 (see Table 1 for details). ECAPs were recorded for different clinical current-levels^2^ (CLs) spanning the patient's dynamic range, sometimes including levels below behavioral threshold. All pulses had 25 μs phase-duration and 58 μs inter-phase gap. For each stimulus condition, raw-ECAPs were recorded in 9 successive sets on 9 recording electrodes. Recording electrodes were the 9 contiguous more-basal electrodes adjacent to the stimulating electrode. Exceptions were subjects S7 and S8, for whom the electrode immediately adjacent to the stimulus electrode was not used for recording (8 recording electrodes).

ECAPs were recorded in 1600 μs epochs on the selected intracochlear recording electrodes (active) with the extra-cochlear electrode MP2 as reference. For each stimulus condition (electrode/current level) and recording electrode, fifty ECAP epochs were successively recorded at a rate of 40 per second and online averaged. Recordings were sampled at 20 kHz and amplified by 50 dB. The recording window started 122 μs after electrical stimulus offset, which was before any ECAP response was expected. Therefore, the voltage of the first raw-ECAP time-sample point (122 μs) of the 1600 μs recording window reflected essentially the electrical artifact.

The ECAP-ICA artifact rejection procedure was as follows. For each stimulus electrode and recording electrode (excluding those on which the amplifier saturated), raw-ECAPs that were obtained from all stimulus levels were concatenated in a random order. The JADE-R Matlab implementation of ICA (Cardoso, 1999) was used, which outputs the mixing matrix from the raw-ECAPs using the desired number of independent components (ICs). The number of ICs for computation was varied between 2 and 9 in order to seek the optimal number of ICs that would explain a sufficient portion of the total raw-ECAP variance without overlearning. Note that Principal Component Analysis (PCA) is a preliminary step that is performed by jade-R in the ICA process to whiten the raw-ECAPs. Therefore, restricting the number of ICs implicitly reduces also the number of PCs. The diagonalized autocorrelation matrix resulting from PCA provided the squared variance of each component on its diagonal. Accordingly, the total amount of variance explained by the number of principal components selected was obtained by summation of the square-root of each component's variance.

Artifact-free ECAPs (ECAP-ICA) were reconstructed on all the recording electrodes using only ECAP sources and replacing the other artifact or noise sources by zeros. Finally, concatenated ECAP-ICAs were segmented into separate time-samples to show individual ECAPs per stimulation level.

### 2.3 ECAP-FM measurement

ECAPs were also measured using the standard forward masking technique (ECAP-FM, Fig. 1) for comparison with ECAP-ICA. The masker pulse was on the same electrode as the probe pulse, had a 10 CL higher current level than the probe pulse, and preceded the probe pulse by 300 μs. The recording electrode was two electrodes more apical (or basal for electrodes 21/22) than the stimulating one. ECAPs were separated from the artifact using the subtraction process described in Fig. 1. All the stimulus parameters and stimulus electrodes were the same as for the ECAP-ICA (see Table 1), including the range of current levels used, but not necessarily the exact CL values.

### 2.4 ECAP analysis

Once ECAPs were obtained from either ICA or FM, they were analyzed to measure their signal-to-noise ratios (SNRs). The recording noise floor was calculated as the root-mean-square (RMS) of the signal after 900 μs, where only noise was expected. The ECAP magnitude (for SNR calculation only) was calculated as the RMS of the signal between 250 and 900 μs. Accordingly, the SNR was the ratio between these two values in decibels. ECAPs were considered as absent if the SNR was lower than 9 dB. This value was chosen based upon initial inspection of the data as the SNR of ‘ECAP’ sources below which they were not consistently visually identifiable as ECAP signals. For SNRs above 9 dB, each ECAP was considered to be present or absent based on visual inspection of the morphology and latency.

ECAP amplitude was measured as the voltage difference between the N_1_ and P_1_ peaks. Accordingly, amplitude growth functions (AGFs) represented the ECAP amplitude in microvolts versus stimulation intensity level in CLs. ECAP morphologies were defined according to a definition by Lai and Dillier (2000): Type Ia for ECAP waveform presenting well-defined N_1_ and P_1_ component; Type Ib for clear P_1_ but no N_1_; Type Ic if clear N_1_ but no P_1_; Type II for ECAPs with two successive peak complexes N_1_P_1_ and N_2_P_2_. ECAP threshold was defined as the intercept between the *x*-axis and the extrapolated linear portion of the ECAP AGF.

## 3 Results

### 3.1 Finding the optimum number of independent components for ECAP-ICA

When applied to the (maximum of) 9 raw-ECAP recording positions, ICA was restricted to a maximum of 9 ICs. Furthermore, it was anticipated that a minimum of three sources would be needed, since there would be one source for each of the stimulus artifact, noise and the ECAP (provided the stimulus was above ECAP threshold). PCA analysis indicated that 4 PCs retained most of the information: more than 97% of the total raw-ECAP variance was explained by the four retained components for all subject and conditions. Note that PC variance does not necessarily indicate the appropriate number of independent components (as these could be correlated with each other), so analysis was completed using different numbers of ICs to determine the optimum number.

**Fig. 2:**
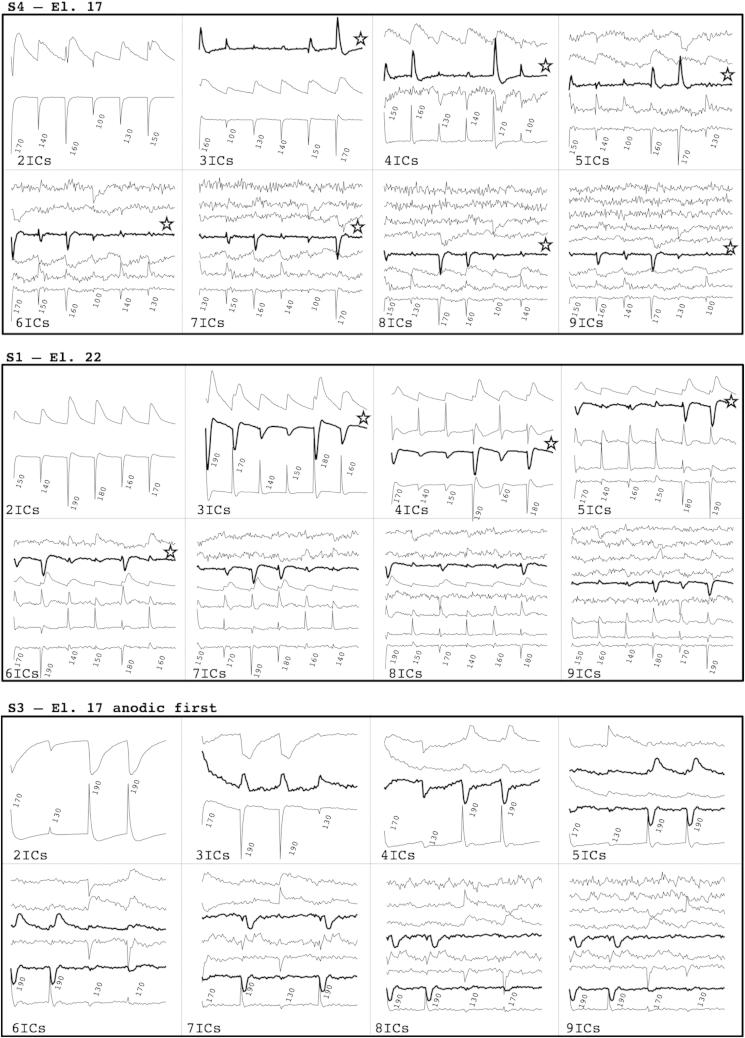
Three examples of the effect of restricting the number of ICs on ICA source projections output to between 2 and 9 ICs. The ECAP source, visually identified, is in bold and identified by a star. For S3 stimulation on electrode 17 anodic-first stimulation, two sources represented the ECAP. This was the only case in which this occurred. Note that the concatenation order for each number of ICs is random and thus different for each number of ICs. Also note that it is difficult to visually distinguish in the figure between neural responses and artifact spikes in the concatenated waveforms. These are distinguished in practice by the latency delays of the neural response which are more obvious in the de-concatonated responses (see Fig. 3). In the bold waveforms here, sometimes artifact spikes occur along with neural responses.

Fig. 2 depicts three examples of limiting the number of components from 2 to 9. For S3 electrode 17, anodic first stimulation, the ECAP signal was split between two ICs when more than 4 ICs were used. Restricting the ICA to two ICs resulted in no visible ECAPs. The ECAP was not separated from the two main artifact components:

• ARTIFACT-SPIKE: a source containing spike-like signals that were similar to raw-ECAPs (sharp exponential decay) and occurred at the beginning of each stimulus epoch even at very low stimulus levels, as expected from stimulation artifact.
• ARTIFACT-LOWPASS: a slow deflexion that took the whole window duration to recover and occurred at each stimulation level like ARTIFACT-SPIKE, suggesting that it was also a non-physiological artifact. Note that this signal was not a side-effect of the concatenation process, as it was also observed in the absence of concatenation. It is possible that it could be an artifact of the measurement process such as caused by amplifier switch-on.

Three 3 ICs were the minimum number of ICs to obtain an identifiable ECAP source. Four ICs resulted in a visible ECAP source for most subjects/electrodes. In that case, the ICs were visually identified as being one of four potential sources: the two artifact sources described above plus the following two sources:

• NOISE: either randomly distributed (Gaussian) recording noise, or residual ARTIFACT-SPIKE that had been split between two different sources (e.g. S4-el.17 in Fig. 2).
• ECAP: a source that reproduced the usual ECAP waveform, such as negative and positive deflexions at appropriate latencies.

**Fig. 3:**
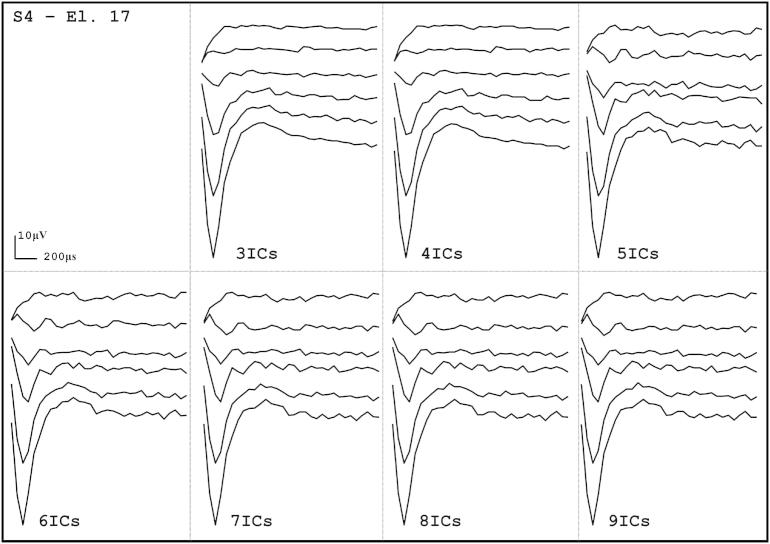
Comparison of ECAP-ICA reconstruction from the ECAP source when different numbers of ICs were used in the ICA.

**Fig. 4:**
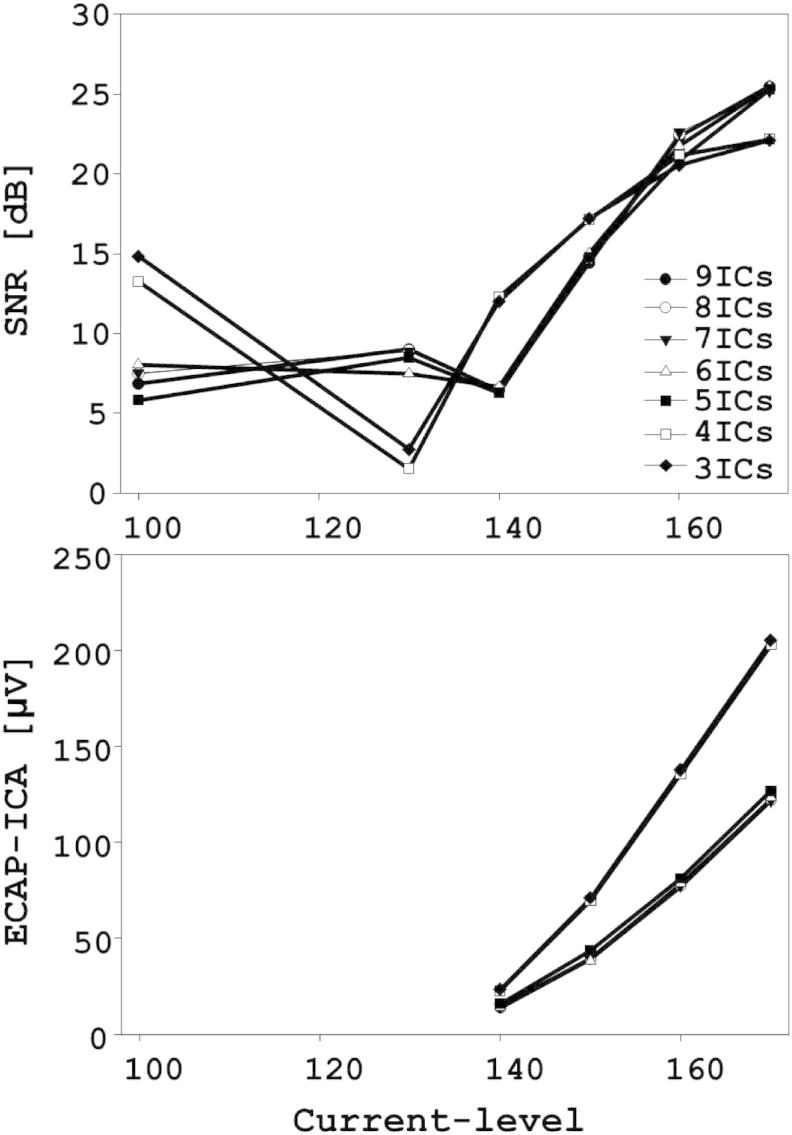
Comparison of SNRs and ECAP-ICA amplitudes when different numbers of ICs were used in the ICA.

When using more than 4 ICs, ICA resulted in the same four types of sources as for 4 ICs plus additional NOISE sources. In addition, the ARTIFACT-SPIKE and ARTIFACT-LOWPASS were more often split between ICs when using more than four ICs. The reconstructed ECAP-ICA waveforms using from 3 to 9 ICs are shown in Fig. 3 for S4-EL17. The SNRs and AGFs derived from the same data are shown in Fig. 4. For this example, the ECAP-ICA waveforms and the SNRs and AGFs were identical with 3 and 4 ICs. They remained very similar although noisier and smaller for more than 5 ICs.

Based on similar analyses across our dataset, the optimal number of ICs to use was determined to be four. A comprehensive description of ICA projection restricted to four ICs is depicted in the supplementary figures for all subjects and conditions. These figures show the sources before deconcatenation of the individual stimulus levels. In these plots, it is sometimes difficult to distinguish by eye artifact spikes, discontinuities due to concatenation at the start of the epochs, and neural responses, as the latency information that distinguishes them is not visible. In practice, neural responses can be distinguished from the spike or concatenation signals by latencies that are non-zero.

With 4 ICs, one condition (S8, EL17) resulted in two ECAP sources, and in this case both sources were retained for the ECAP reconstruction. In 8 further cases, as well as main ECAP source, there was an indication of a small residual ECAP component in a non-ECAP source, leading to the procedure discarding that ECAP portion in the reconstruction phase. In six of these latter cases, the problem occurred only at a specific current level (usually a high one), and this would not have greatly affected any measure of ECAP-ICA threshold using this method. These cases included S1 on E14 and E17, S2 and S7 on EL 17, S4 on EL22, and S3 on EL17-anodic first. In two cases (S1 on E21, and S5 on E22) the problem occurred at more than one stimulus level. Overall, 4 ICs provided the best compromise between successful isolation of the ECAP and the danger of splitting the ECAP response into more than one IC. The further analysis presented below used the outcomes of ICA using 4 ICs.

### 3.2 ECAP-ICA and artifact AGFs

**Fig. 5:**
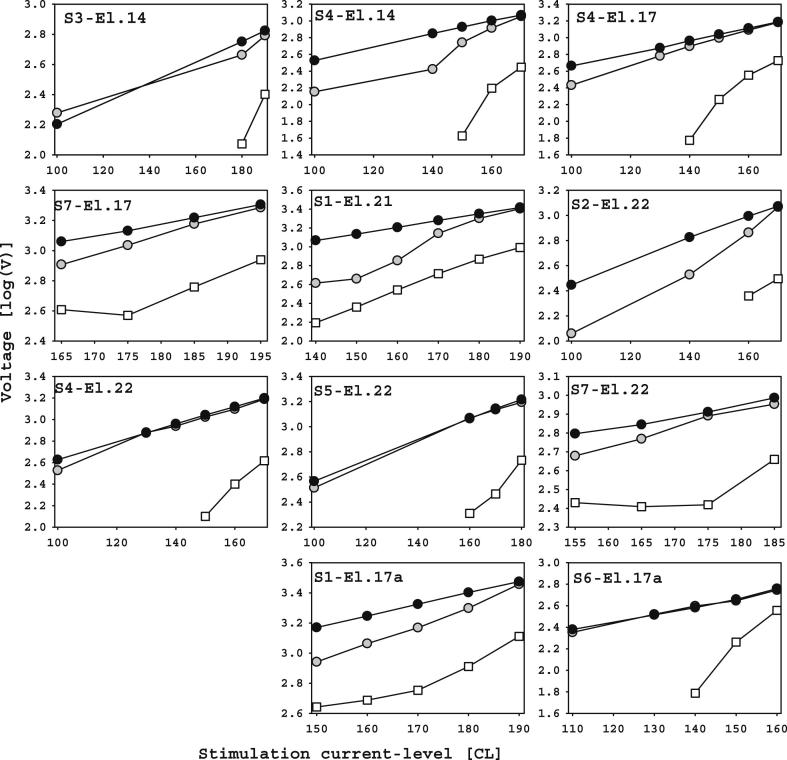
ECAP-ICA (white), artifact (grey) and raw-ECAP (black) AGFs on a fixed recording electrode.

To verify ECAP-ICA validity, the ECAP-ICA and artifact AGFs were compared (Fig. 5). The reconstructed ARTIFACT-SPIKE amplitude was determined as the maximum (always first) voltage measure of the reconstructed ARTIFACT-SPIKE source, which was calculated by setting all sources except ARTIFACT-SPIKE to zero. When the spike artifact was split between two ICs (the NOISE source sometimes contained residual artifact spike), it was not possible to use the amplitude of the reconstructed ARTIFACT-SPIKE for comparison with the ECAP-ICA amplitude. However, the first time-sample of the raw-ECAP should be dominated by the stimulus artifact, since its latency is earlier than any expected physiological response, and so provides an alternative measure of artifact amplitude. Fig. 5 shows both the raw-ECAP amplitude (black), and the reconstructed ARTIFACT-SPIKE amplitude (gray), for subjects/electrodes where the spike artifact was not split between ICs, and also 4 cases in which it was split, but the ARTIFACT-SPIKE reconstructed source showed a similar AGF pattern to the raw-ECAP AGF. The latter cases (S3 and S4 El14, S1 El21and S2 El22), except for S3 EL14, show the largest discrepancy in Fig. 5 between the two measures of artifact amplitude as expected. The open symbols in Fig. 5 are the ECAP amplitude (N1-P1) for those conditions in which it was determined that ECAP was present (SNR >=9 dB together with correct latency and morphology). The influence of ARTIFACT-LOWPASS was disregarded in the comparisons of amplitudes in this section.

Over the selected dataset, the artifact amplitude (derived from the reconstructed signal from ATIFACT-SPIKE) and raw-ECAP amplitudes (first measure of the recording epoch) were usually very similar and, the ECAP-ICA amplitude was generally around an order of magnitude lower than that of both artifact and raw-ECAP. Importantly, the slopes of the ECAP and ARTIFACT-SPIKE AGFs differed, and when below-threshold currents were used, it was clear that artifacts remained below ECAP threshold.

**Fig. 6:**
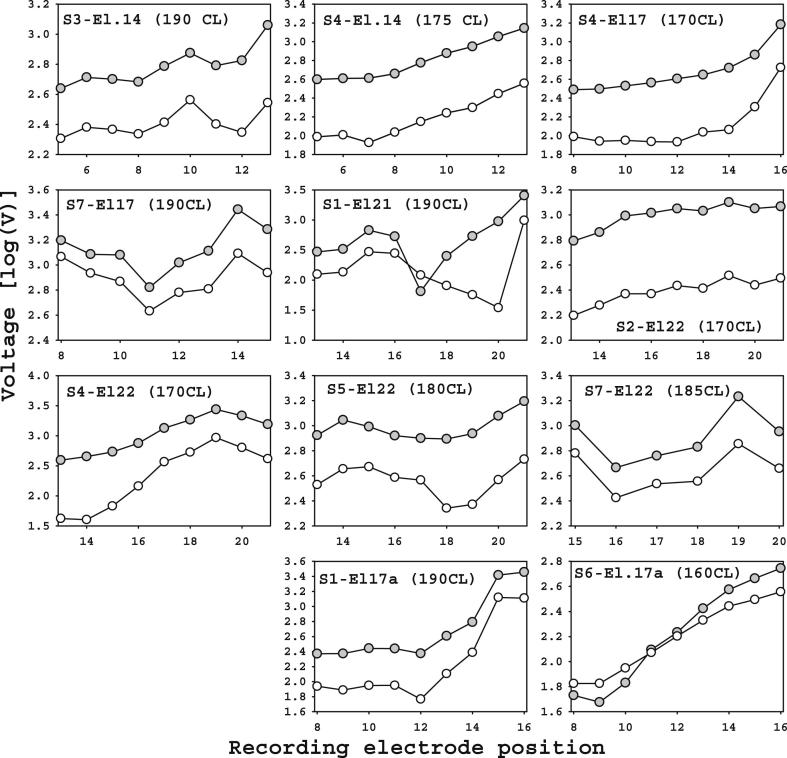
ECAP-ICA (white) and artifact (grey) amplitudes on different recording electrodes for a fixed stimulus current-level.

Fig. 6 shows how the ECAP-ICA and artifact amplitudes varied across recording electrode positions. The way that ECAP and artifact amplitudes varied with recording electrode position was highly correlated, with the ECAP generally around an order of magnitude lower than the artifact. However, subtle differences in effect of recording position occurred, which were important for ICA to efficiently separate artifact and ECAP.

### 3.3 ECAP-ICA compared to ECAP-FM

**Fig. 7:**
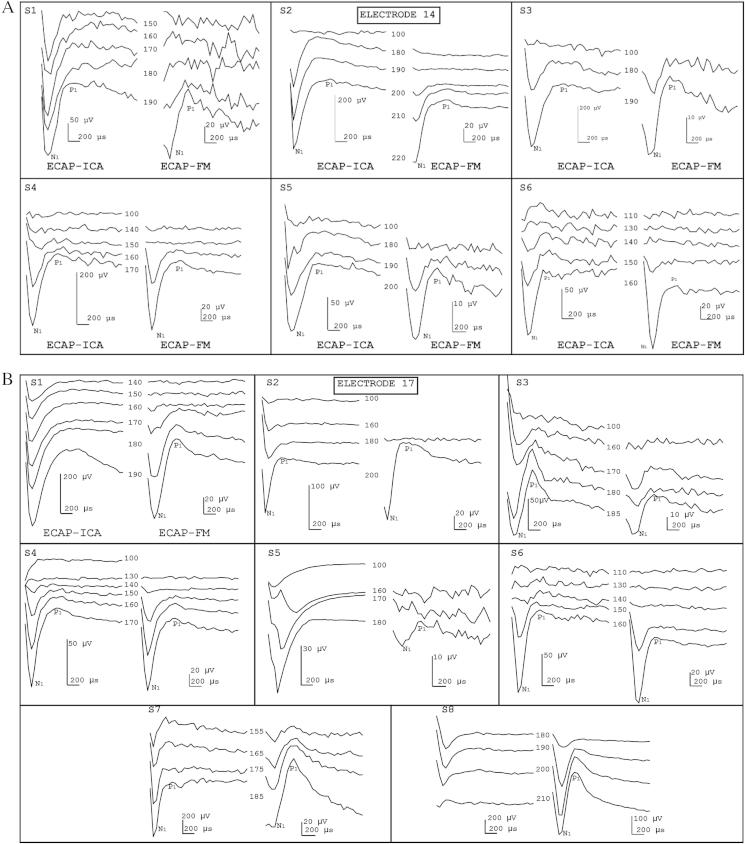

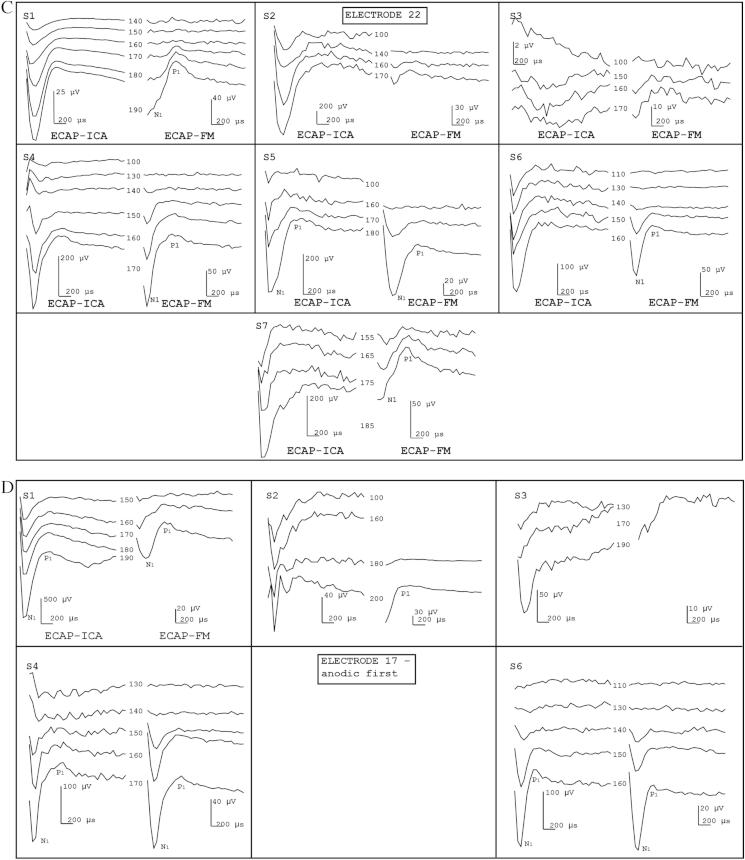
ECAP-ICA compared to ECAP-FM. In each panel, the ECAP-ICA waveforms reconstituted from the ECAP source are plotted for different stimulation current levels and compared to the waveforms obtained from ECAP-FM (ECAP-ICA on the left and ECAP-FM on the right). (A) Stimulation on electrode 14; (B) Stimulation on electrode 17; (C) Stimulation on electrode 22. Note that electrode 21, instead of 22, was used for S3 and S6; (D) Stimulation on electrode 17 anodic-first polarity.

In the absence of an ‘ECAP gold-standard’ for cochlear implants, the most common method used clinically (ECAP-FM) was used to compare with ECAP-ICA. ECAP-FM and ECAP-ICA waveforms can be visually compared for each stimulation condition tested in Fig. 7A to D. If both measures arise from an identical neural response, then it would be expected that the latencies and waveform morphologies should be correlated with each other, if not identical. However as mentioned in Section 1.2, ECAP-FM waveforms may be distorted, and the ECAP-ICA signal could be distorted by non-perfect separation of ECAP from artifact and noise. Therefore, it was expected that differences may exist.

The reconstructed ECAP-ICA was usually sign-inverted compared to ECAP-FM, with the exception of two cases (S7 and S8 on electrode 17). However, the polarity of the response was always consistent across the concatenated stimulus-level conditions. Furthermore it can be seen in Fig. 7 that the ECAP-ICA amplitude was sometimes significantly greater than ECAP-FM amplitude. The variation in reconstructed source polarity and differences in amplitude are likely to be a result of scale and sign ambiguities inherent in ICA analysis. Naik and Kumar (2011) have demonstrated mathematically that, since the actual variance of the sources is unknown, an inherent scale ambiguity arises, since any linearly scaled version of the sources would fit the model equally. In practice, the analysis sets all the variances of the sources to unity, and this assumption of equal variance is not likely to be valid in our particular case. This ambiguity does not affect the use of the ICA method to determine ECAP thresholds, for example, as all the ECAPs in the concatenated waveform (from different stimulus levels), as well as other sources, are scaled by the *same* unknown scaling factor. Thus different scaling factors would produce the same AGF when the AGF is normalised relative to (say) the maximum amplitude.

Another possibility that should be considered is that the ECAP-ICA sometimes contained some residual artifact. Depending on the relative polarity of the artifact and ECAP response, and the time-course of the artifact decay, any remaining artifact present at the ECAP peak latencies could either enhance or reduce the N1P1 amplitude. However the presence of significant artifact would be evident in the ECAP waveforms as it would impose a significant additional slope across the measurement window. Comparing the waveforms in Fig. 7, it does not appear that the ECAP-ICA waveforms are systematically different in general shape from the ECAP-FM waveforms. In particular there is no evidence of residual artifact near time zero in the low-stimulus-level conditions below ECAP threshold.

As expected, ECAP-FM and ECAP-ICA showed a similar range of morphologies. All types of previously reported ECAP waveform morphologies were seen in our dataset. Table 2 shows the morphology categories for ECAP-FM and ECAP-ICA. It can be seen that the same morphology type was obtained for individual ICA and FM in half of the 26 cases.

The ECAP-ICA peak latencies were measured for comparison with those of the ECAP-FM measured here and with ECAP-FM latencies reported in the literature. We have compared the N1 latencies only, as the P1 latencies were often difficult to precisely determine. N1 latencies of ECAP-ICA occurred in the normative range of 220–470 μs (defined by Cafarelli Dees et al., 2005). ECAP-FM and ECAP-ICA N_1_ latencies both ranged from 245 μs (S2 on El.17 for both FM and ICA) to 349 μs (S1 on El.22 for FM and ICA, S2 on El.22 for ICA, S5 on El.22 for FM and S6 on El. 14 and 17 for FM). Median N_1_ latencies for ECAP-FM and ECAP-ICA were 309 and 301 μs respectively, and their distributions did not differ significantly (Mann–Whitney *U* = 185, *N*_ECAP-FM_ = 21 and *N*_ECAP-ICA_ = 22, *p* = 0.263). Pearson correlation moment between the latencies was not significant (*p* = 0.831), possibly reflecting the small range of latency values from each method.

**Fig. 8:**
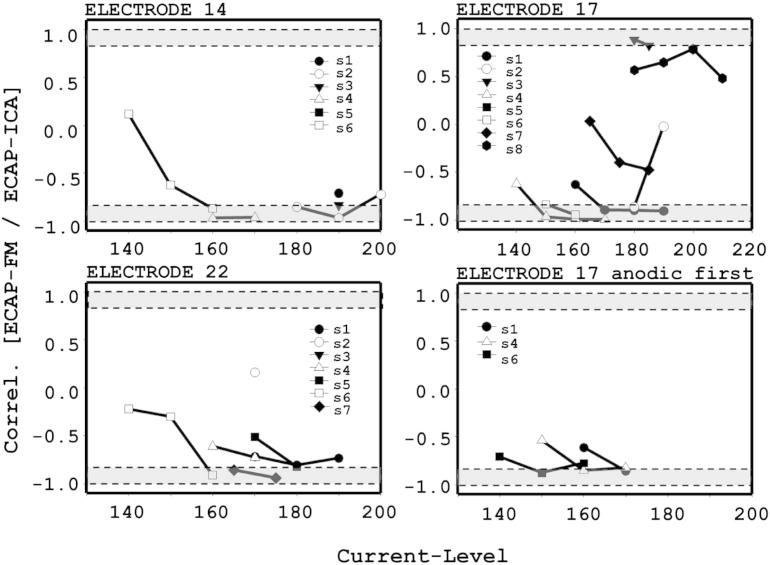
ECAP-FM and ECAP-ICA waveform correlations. For each stimulation electrode, correlation coefficients are plotted as a function of stimulus level. The grey areas highlight correlation coefficients with magnitude higher than 0.8 - i.e. strong similarity between ECAP-FM and ECAP-ICA. A negative co-efficient signifies that the ECAP-ICA waveform was inverted relative to the ECAP-FM waveform. Note that electrode 21, instead of 22, was used for S3 and S6.

To further quantify the overall resemblance of ECAP-FM with ECAP-ICA waveforms, cross-correlation coefficients (*R*) were calculated. This analysis was restricted to well-defined ECAPs only (with SNR higher than 9 dB). The results are summarised in Fig. 8. All correlations were statistically significant (*p* < 0.05). At the highest current-level, 60% of the calculated |*R*|-values were greater than 0.8 in spite of subtle variations in morphology. In one case only (S2 on El 22) was the |*R*|-value smaller than 0.2. Note that negative correlation coefficients corresponded to ECAP-ICA with opposite polarity to ECAP-FM.

**Fig. 9:**
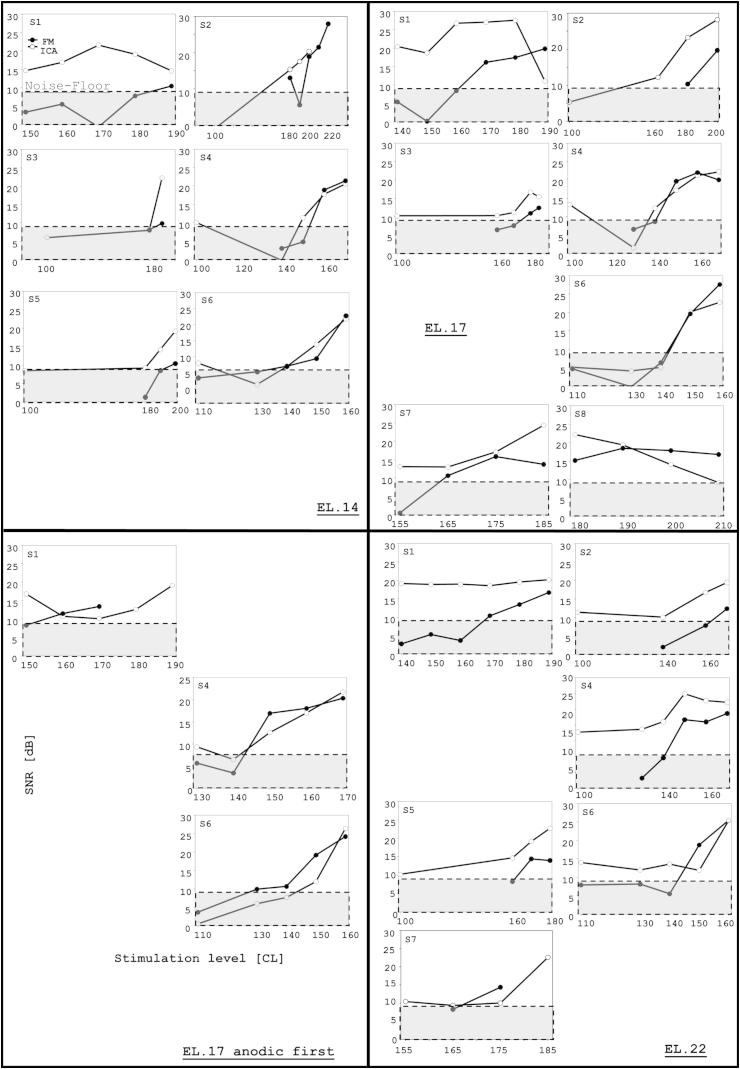
Signal-to-noise ratio of ECAP waveforms as a function of stimulus level. Filled circles represent ECAP-FM, and open circles represent ECAP-ICA. The grey area represents SNRs below 9 dB, where it was considered that ECAPs were not clearly different from recording noise. Each panel represents a different stimulation electrode. All *x*-axes are graduated in current-levels and *y*-axes in dB, as shown once in the bottom-left panel. Note that electrode 21, instead of 22, was used for S6.

Fig. 9 shows the SNR values for each condition (including all stimulus levels whether or not ECAP was present). The shaded area in the figure highlights the data that fell in the SNR <9 dB area, where ECAP was deemed to be absent. Note that some points where SNR was greater than 9 were conditions in which ECAP was also absent, based upon visual inspection of morphology and latency. SNRs were significantly larger for ECAP-ICA than for ECAP-FM on electrode 14 (*t* = 2.22, *p* = 0.03) and electrode 22 (*t* = 4.09, *p* < 0.001). The difference failed to reach significance for electrode 17 (*t* = 1.695, *p* = 0.09).

**Fig. 10:**
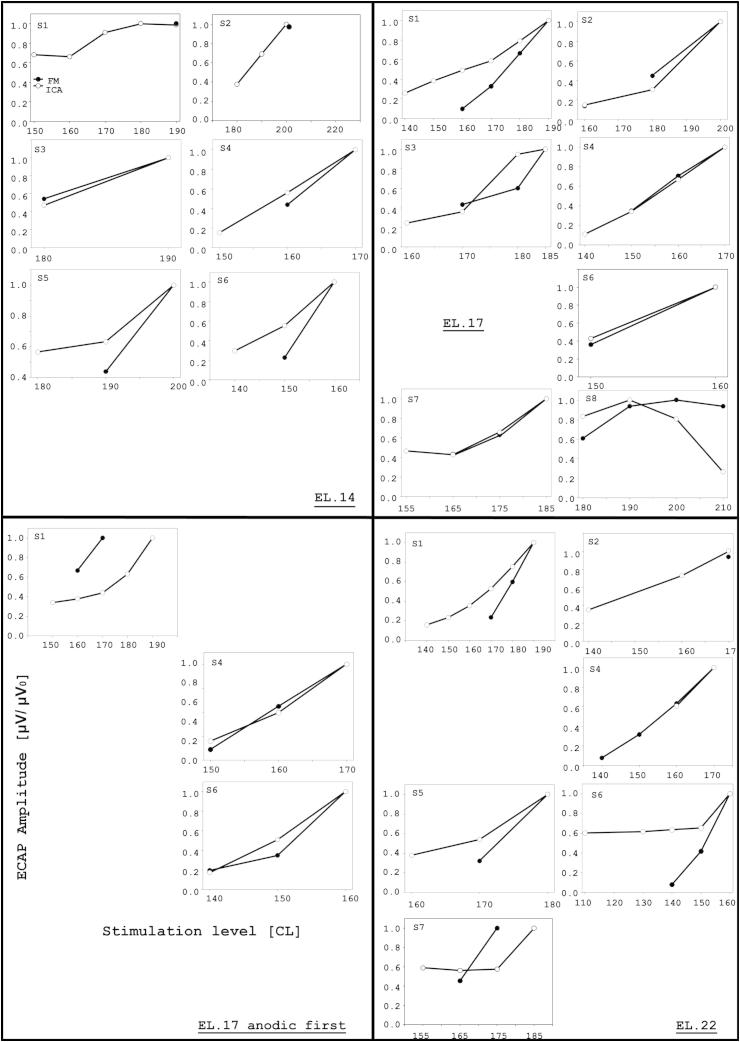
Normalized ECAP-FM and ECAP-ICA AGFs. Only ECAPs with SNR >9 dB were plotted. Filled circles represent ECAP-FM, and open circles represent ECAP-ICA. All *x*-axes were graduated in current-levels and *y*-axes in normalised amplitudes (μV/μV_0_), as shown in the bottom-left panel. Note that electrode 21, instead of 22, was used for S6.

ECAP-ICA and ECAP-FM AGFs are compared in Fig. 10. To compare the shape of the functions, both were normalised to the maximum amplitude obtained for each function. As expected, different AGF patterns were observed for different subjects and electrodes, but also between ECAP-FM and ECAP-ICA. ECAP-FM tended to produce relatively linear growth functions for all subjects except S8 (EL17), for whom an upper plateau was observed for levels above 190 CL. Similarly, ECAP-ICA amplitude generally increased linearly with stimulation level; however these linear portions were sometimes preceded by an initial part with a shallower slope. An exception was again S8 (EL17), whose ECAP-ICA amplitude decreased with increasing current level above 190 CL. It should be noted that the SNR for the ECAP-ICA was also decreasing with increasing current level (Fig. 9) for S8, and that S8 (EL17) was the condition in which ECAP was very clearly split between two sources (see supplementary Figure B). In this case it is likely that some of the ECAP was being discarded in the artifact or noise sources for this subject, as the ICA procedure failed to separate sources effectively.

### 3.4 Discussion

The three criteria for ECAP-ICA validity proposed in the introduction were satisfied in most cases. Despite a similar overall pattern, the artifact and ECAP-ICA amplitudes changed differently along the recording electrodes, allowing these sources to be successfully separated in the majority of cases using ICA. This observation would confirm the intuitive idea that a certain minimal number of recording points (above the theoretical minimum of 4 for the four sources) are necessary so that ICA can consider ECAP and artifact as independent sources. Secondly, the ECAP-ICA and artifact AGFs had different slopes and different intercepts in those cases where we used stimuli below ECAP threshold, supporting the proposition that the physical sources of these two derived potentials were independent. Thirdly, the ranges of waveforms and latencies of ECAP-ICA were consistent with normative data reported in the literature and measured here using ECAP-FM (Cafarelli Dees et al., 2005; Lai and Dillier, 2000).

The results confirmed the intuitive biophysical consideration that AN responses (ECAPs) and the electrical stimulation (artifact) signals may be considered as independent signals, especially from the point of view of an intracochlear recording electrode. The artifact and the ECAP potentials arise from two distinct locations, and in surrounding biological tissues that differ in impedance. The relation between ECAP and artifact potentials is likely to change at different recording electrode positions along the curvature of the modiolus, resulting in a non-linear relationship between the artifact and ECAP signals recorded on the electrodes.

An important feature of our ECAP-ICA procedure was the concatenation of raw-ECAPS for different stimulus levels. The inclusion of stimulus levels that induced artifact without neural response helped to make these two sources less statistically dependent. A potential improvement of the technique might be obtained by similar strategies, for example the inclusion of some masked stimulus pulses in the concatenation, for which the artifact and neural response would have a different relationship than for the unmasked pulses.

We arbitrarily used nine (or eight) recording electrodes in the example data, which was probably more than necessary. It seems reasonable to include at least several more than four recording locations in order to increase the chances of including recordings where ECAP and artifact amplitudes behave independently and to allow for amplifier saturation or anomalous recordings. The optimisation of the number and position of recording electrodes is the aim of a future study.

An advantage of ICA is that neither masker, nor alternate polarity stimuli are needed. ECAP-ICA aims to avoids the biases (underestimation or distortion of ECAP) produced by forward-masking and alternate polarity methods. However, the ICA method also has intrinsic drawbacks. Most importantly, ECAP-ICA may also underestimate ECAP amplitude if ICA doesn't fully separate the ECAP into a single source, or overestimate the amplitude if artifact remains in the ECAP source. It should be remembered that the absolute amplitude derived by ICA is subject to an arbitrary linear scaling, so only the relative amplitude differences for different conditions in the concatenated data are relevant. It is also possible that ECAP-ICA thresholds may be lower than ECAP-FM thresholds on average, since ECAP amplitude underestimation due to incomplete masking may occur in the ECAP-FM case, and the SNR was, on average, better with ECAP-ICA. However, it was not possible to assess differences in ECAP thresholds here, given the limited number of points collected in the AGFs and the limited number of electrodes/subjects compared.

ECAP-ICA can be measured over a greater perceptual dynamic range than ECAP-FM. The use of a masker with a higher level than the probe in the forward-masking technique imposes an upper comfortable limit on the probe current level used that is lower than the upper limit for ECAP-ICA. This is both because the masker has a higher current than the probe, and also because the masker and probe together produce a loudness greater than either alone due to temporal integration.

The ICA technique may be improved in speed, efficiency and accuracy by ECAP recording technology that allows simultaneous recordings. Simultaneous recording technology is not yet available, however. At the moment, ECAP-ICA requires nine sequential buffers to be recorded, whereas forward-masking requires only four and alternate-polarity two. With simultaneous recordings, the nine ECAP-ICA buffers could be obtained simultaneously, reducing the time to one ninth of that used in this experiment, whereas the buffers in ECAP-FM correspond to different stimulus conditions and so cannot be made simultaneously. The use of sequential recordings could lead to a distorted or diminished ECAP waveform if substantial neural adaptation occurred over the multiple successive recordings. Theoretically, the use of simultaneous recordings would make it easier than in the sequential recording case to separate Gaussian noise from the artifact and neural response, since the noise would be correlated across recording positions in the simultaneous case only. The level of noise was quantified in this study as an SNR, rather than an absolute level (designated as any signal under 50 μV by Lai and Dillier (2009) for the CI24RE cochlear implant or under 20 μV by Cafarelli Dees et al. (2005) for the CI24R). Although the SNRs were, on average larger for ECAP-ICA than for ECAP-FM, this may not be necessarily due to better removal of Gaussian noise in the ICA process. In both cases, noise reduction was first achieved by averaging 50 epochs in each condition. In ECAP-FM (and potentially in ECAP-ICA) any Gaussian noise remaining will appear in the final ECAP.

## 4 Conclusions

The ICA-based artifact cancellation for ECAP successfully allowed the measurement of ECAP responses without the need for masker pulses or the use of alternate polarity. ECAP-ICA and ECAP-FM had a similar range of waveform morphologies and the same range of peak latencies, even though differences were seen in individual conditions. These differences were probably due to different violations in either technique's assumptions. For example, it is possible that masking of the probe response was incomplete in ECAP-FM leading to underestimation of the ECAP amplitude and/or distortion of morphology; and for ECAP-ICA, the ECAP response may potentially be split between two sources and thus partially discarded.

## Figures and Tables

**Table 1:** Subject information.

Subject ID	Age (yr)	Profound deafness duration (yr)	Etiology	Duration of CI use (years)	Implantation side	Conditions tested(electrode and polarity)
S1	52	7	Familial progressive	3	Right	14, 17, 22 cathodic first
						17 anodic first
S2	63	7	Viral infection	4	Right	14, 17, 22 cathodic first
						17 anodic first
S3	65	12	Idiopathic progressive	3	Right	14, 17, 21 cathodic first
						17 anodic first
S4	52	20	Congenital progressive	4	Left	14, 17, 22 cathodic first
						17 anodic first
S5	65	20	Idiopathic progressive	5	Left	14, 17, 22 cathodic first
S6	75	5	Chronic suppurative otitis media	3	Right	14, 17, 21
						17 anodic first
S7	64	Unknown	Familial progressive	5	Right	17, 22 cathodic first
S8	49	5	Idiopathic sudden	4	Right	17 cathodic first

**Table 2:** ECAP waveform morphology. Underlined subjects were those who showed similar waveforms for ECAP-ICA and ECAP-FM.

	Electrode 14		Electrode 17		Electrode 17 anodic 1st		Electrode 22	
	ECAP-ICA	ECAP-FM	ECAP-ICA	ECAP-FM	ECAP-ICA	ECAP-FM	ECAP-ICA	ECAP-FM
TYPE I-a	S4	S1,S2,S3, S4,S6	S1,S3,S4, S6	S1,S2,S3, S4,S6, S8	S1,S4,S6	S1,S4,S6	S1,S4,S6	S2,S4,S5, S6
TYPE I-b	–	–	–	S7	–	S2	–	S1,S7
TYPE I-c	S1,S2,S3, S5,S6	S5	S2,S8	S5	S3	–	S2,S6	–
TYPE II	–	–	S7	–	S2	–	S7	–
No ECAP	–	–	S5	–	–	S3	S3	S3

## Appendix A Supplementary data

Detail of ICA output obtained for the complete dataset. The following is the supplementary data related to this article:

Supplementary figure: Raw-ECAPs and independent components outcome of ICA. In each panel the four sources extracted by ICA are shown, together with the raw-ECAP from all recording channels superimposed (depicted below the dashed lines). The ICs are labelled with the source identification assigned by visual inspection (A-S, ARTIFACT-SPIKE; N-S, NOISE (Gausian or residual spike); A-LP, ARTIFACT-LOWPASS). The whole dataset was derived from analysis with 4ICs. (A) Stimulation on electrode 14, (B) Stimulation on electrode 17, (C) Stimulation on electrode 22 (Note that electrode 21, instead of 22, was used for S3 and S6), and (D) Stimulation on electrode 17 anodic-first polarity. The corresponding reconstructed ECAP-ICAs obtained are plotted in Figures 7A to 7D

## Footnotes

2 For CI24RE cochlear implants, 1CL = 0.157 dB step. **1***Akhoun, McKay and El-deredy – ECAP-ICA*.
