# Supplementary material for: Electrically evoked compound action potential artifact rejection by independent component analysis: Technique validation
Source: Hear Res. 2013 Aug;302:60–73. doi: 10.1016/j.heares.2013.04.005 (PMC3709093; doi:10.1016/j.heares.2013.04.005)
Supplement: Supplementary file 1 — Supplementary figure: Raw-ECAPs and independent components outcome of ICA. In each panel the four sources extracted by ICA are shown, together with the raw-ECAP from all recording channels superimposed (depicted below the dashed lines). The ICs are labelled with the source identification assigned by visual inspection (A-S, ARTIFACT-SPIKE; N-S, NOISE (Gausian or residual spike); A-LP, ARTIFACT-LOWPASS). The whole dataset was derived from analysis with 4ICs. (A) Stimulation on electrode 14, (B) Stimulation on electrode 17, (C) Stimulation on electrode 22 (Note that electrode 21, instead of 22, was used for S3 and S6), and (D) Stimulation on electrode 17 anodic-first polarity. The corresponding reconstructed ECAP-ICAs obtained are plotted in Figures 7A to 7D [file mmc1.docx]

Supplementary Figure A
