# Supplementary material for: Electrically evoked compound action potential artifact rejection by independent component analysis: Technique validation
Source: Hear Res. 2013 Aug;302:60–73. doi: 10.1016/j.heares.2013.04.005 (PMC3709093; doi:10.1016/j.heares.2013.04.005)
Supplement: Supplementary file 3 [file mmc3.doc]

Supplementary Figure C
